# Phylogenetic diversity and the structure of host-epiphyte interactions across the Neotropics

**DOI:** 10.7717/peerj.15500

**Published:** 2023-06-19

**Authors:** Marcio R. Pie, Fernanda S. Caron, Thom Dallimore, Helena Einzmann, Peter Hietz, Michael Kessler, Flavio Nunes Ramos, João Pedro Costa Elias, Holger Kreft, Thorsten Krömer, Maria Judith Carmona Higuita, Daniel Zuleta, Giesta Machado, André Luís de Gasper, Gerhard Zotz, Glenda Mendieta Leiva, Derio Antonio Jimenez-Lopez, Alex Fernando Mendes, Pedro Brancalion, Sara Mortara, Christopher Thomas Blum, Mariana Victória Irume, Nayely Martínez-Meléndez Nayely, Ana Maria Benavides, Carlos Renato Boelter, Sven Batke

**Affiliations:** 1 Biology Department, Edge Hill University, Ormskirk, United Kingdom; 2 Departamento de Zoologia, Universidade Federal do Paraná, Curitiba, Brazil; 3 World Museum, National Museums Liverpool, Liverpool, United Kingdom; 4 Institute for Biology and Environmental Sciences, Carl von Ossietzky Universität Oldenburg, Oldenburg, Germany; 5 Institute of Botany, University of Natural Resources and Life Sciences, Vienna, Austria; 6 Department of Systematic and Evolutionary Botany, University of Zurich, Zurich, Switzerland; 7 Instituto de Ciências da Natureza, Universidade Federal de Alfenas, Alfenas, Brasil; 8 Biodiversity, Macroecology & Biogeography, University of Göttingen, Göttingen, Germany; 9 Universidad Veracruzana, Xalapa, Mexico; 10 Forest Global Earth Observatory, Smithsonian Tropical Research Institute, Washington DC, United States of America; 11 Departamento de Ciências Naturais, Universidade Regional de Blumenau, Blumenau, Brazil; 12 Institute for Biology and Environmental Sciences, Carl von Ossietzky University, Oldenburg, Germany; 13 Smithsonian Tropical Research Institute, Balboa, Panama; 14 Phillips University of Marburg, Marburg, Germany; 15 Programa de doctorado en Ciencias, El Colegio de la Frontera Sur, San Cristóbal de las Casas, Chiapas, Mexico; 16 Departamento de Ciências Florestais, Escola Superior de Agricultura Luiz de Queiroz, Universidade de São Paulo, São Paulo, Brazil; 17 Universidade de São Paulo, São Paulo, Brazil; 18 International Institute for Sustainability IIS-Rio, Rio, Brazil; 19 Universidade Federal do Paraná, Curitiba, Brazil; 20 Coordenação de Biodiversidade, Instituto Nacional de Pesquisas da Amazônia - INPA, Amazônia, Brazil; 21 Ministry of Environment and Natural History, Orquidario and Botanical Garden “Comitán”, Chiapas, Mexico; 22 Medellin Botanical Garden, Medellín, Colombia; 23 Centro Zamorano de Biodiversidad, Departamento de Ambiente y Desarrollo, Escuela Agricola Panamericana, Francisco Morazan, Honduras

**Keywords:** Evolution, Commensalism, Neotropics, Trees, Forests, Distinctiveness

## Abstract

Understanding the mechanisms driving community assembly has been a major focus of ecological research for nearly a century, yet little is known about these mechanisms in commensal communities, particularly with respect to their historical/evolutionary components. Here, we use a large-scale dataset of 4,440 vascular plant species to explore the relationship between the evolutionary distinctiveness (ED) (as measured by the ’species evolutionary history’ (SEH)) of host species and the phylogenetic diversity (PD) of their associated epiphyte species. Although there was considerable variation across hosts and their associated epiphyte species, they were largely unrelated to host SEH. Our results mostly support the idea that the determinants of epiphyte colonization success might involve host characteristics that are unrelated to host SEH (e.g., architectural differences between hosts). While determinants of PD of epiphyte assemblages are poorly known, they do not appear to be related to the evolutionary history of host species. Instead, they might be better explained by neutral processes of colonization and extinction. However, the high level of phylogenetic signal in epiphyte PD (independent of SEH) suggests it might still be influenced by yet unrecognized evolutionary determinants. This study highlights how little is still known about the phylogenetic determinants of epiphyte communities.

## Introduction

The study of community assembly mechanisms has a long tradition in ecology that has provided valuable insights into the organization of ecological communities (Gleason, 1927; Clements, 1936; Diamond, 1975; Fukami, 2015). Nevertheless, an evolutionary perspective has only been added recently (Emerson & Gillespie, 2008; Narwani et al., 2015). Mutualistic, and/or parasitic community relationships have been intensively studied in animals and plants (Rezende et al., 2007b; Krasnov, Poulin & Mouillot, 2011), however, commensal communities have been overlooked. Most likely, the scarcity of studies on commensal organisms might be due to the opportunistic nature of their interactions. Indeed, that property could suggest that these interactions are less affected by processes at evolutionary timescales.

Regardless of the type of interaction, there are two main approaches to study community assembly in an evolutionary perspective: phylogenetic diversity and community phylogenetics (Faith, 1992; Webb et al., 2002). Phylogenetic diversity (PD) is a measure of the total amount of phylogenetic distance between species in a community (Faith, 1992). Throughout the text we refer to PD as the general concept of describing the phylogenetic history present in a set of taxa, rather than the specific metric proposed by Faith (1992). Although PD was initially seen as a tool for conservation prioritization, later studies have found that PD plays a role in ecosystem stability (Cadotte, Dinnage & Tilman, 2012) and might also be used to predict ecosystem functions (Srivastava et al., 2012; Cadotte, 2015). Community phylogenetics (Webb et al., 2002; Swenson et al., 2006) on the other hand, seeks to understand how the phylogenetic relationships of sets of local species might reflect sorting mechanisms. These sorting mechanisms can include interspecific competition or environmental filtering that act on their regional species pools (Pearse et al., 2014; Kraft et al., 2015). Although these two approaches are clearly related, they provide complementary views on the role of historical processes on community organization. Even though these concepts are complementary, they tend to be used in different contexts through the scientific literature and are rarely explored in the same study.

Here we explore patterns of PD in vascular epiphytes and their hosts. Epiphytes are plants that grow on other plants for physical support but are not parasitic (Benzing, 2008; Zotz, 2013a; Zotz, 2013b). They contribute about 10% of global vascular plant diversity (Zotz et al., 2021b) and account for up to 23–27% of the diversity of plants regionally, or nationally, across the Neotropics (Cascante-Marín & Nivia-Ruíz, 2013; Batke, Cascante-Marin & Kelly, 2016). Epiphytes are most diverse in tropical regions, especially in the Neotropics (Zotz, 2005; Suissa, Sundue & Testo, 2021) and there are extreme cases of high abundance where they can cover most of the surface area of the host tree. Hundreds of papers have been published since the late 1800s describing the ecology, biogeography, taxonomy, and population genetic structure of epiphytes (Zotz & Hietz, 2001; Zotz, 2013a; Menini Neto et al., 2016; Zotz, Hietz & Einzmann, 2021c; Chaves et al., 2021; Taylor et al., 2022; Marcusso et al., 2022). Many studies have related within and between canopy epiphyte assemblages to environmental and structural properties of the host tree (Morales-Linares et al., 2020; Victoriano-Romero et al., 2020) and also focused more recently on epiphyte-host tree commensalistic networks (Burns, 2007; Burns & Zotz, 2010; Silva et al., 2010; Piazzon, Larrinaga & Santamaría, 2011; Ceballos, Chacoff & Malizia, 2016; Francisco et al., 2018; Francisco et al., 2019; Naranjo et al., 2019; Zotarelli et al., 2019; Cortés-Anzúres et al., 2020). Some studies have shown that host-tree characteristics can be important in local epiphyte assemblages (see Wagner, Mendieta-Leiva & Zotz, 2015), however, we do not know whether host PD is a suitable predictor of epiphyte PD. However, our understanding of the drivers of epiphyte PD is sorely limited, with only two studies published to date, with only one investigating the relationship between host and epiphyte PD. For example, the study by Kluge & Kessler (2011) focused specifically on the phylogenetics of epiphytic fern communities (but not host species) in two locations in Costa Rica and found evidence of phylogenetic clustering that was consistent with environmental filtering in stressful conditions (drought at low elevations and frost at high elevations), whereas milder conditions were associated with overdispersion, possibly due to interspecific competition and character displacement. The only study that investigated host and epiphyte PD in a coastal area of Veracruz, Mexico, found that perturbation did not have an effect (Aguirre et al., 2010). However, they found that palms harbored a more phylogenetically diverse epiphyte community than other host trees. However, this study was limited taxonomically and geographically (a total of nine families, 16 genera and 21 species of epiphyte), making generalisation regarding the phylogenetic component of host-epiphyte associations difficult.

**Figure 1:**
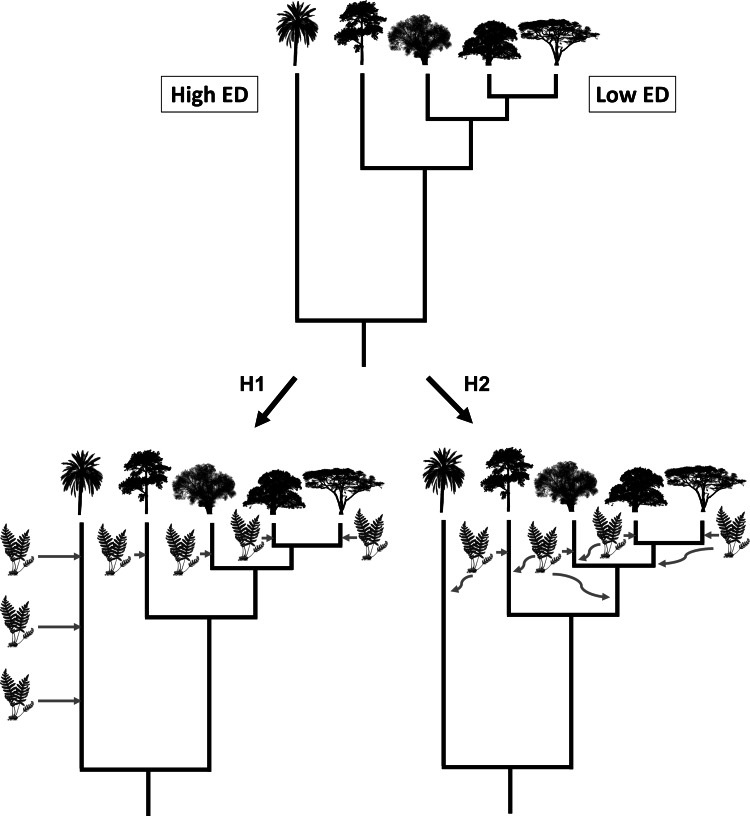
Visualisation of hypothesis one (H1) and two (H2). The silhouette of the fern represents epiphyte species and the tree silhouettes different linages of trees. ED refers to evolutionary distinctiveness.

Here, we use a large-scale dataset of 4,440 species of vascular epiphytes and host plants to test three alternative hypotheses regarding epiphyte PD and their host trees. First, a host tree that is evolutionarily unique might possess ecological and structural characteristics that are uncommon (*e.g.*, very acute branch angles or flaky and shedding bark), which might hamper the colonization of epiphytes, leading to a low PD of its associated epiphyte communities. Likewise, host species with low evolutionary uniqueness would tend to share characteristics with other co-occurring species, thus allowing for the same epiphyte assemblages to colonize many host species. Our first hypothesis is that this scenario would result in a negative relationship between epiphyte PD and host plant evolutionary distinctiveness (ED) (Fig. 1, see the methods section below for a more precise definition of PD and ED). Our second hypothesis is that an evolutionarily unique host plant would have been available for colonization for a long time, allowing ample time for epiphytes to be selected to colonize it by natural selection, whereas relatively young lineages could show novel adaptations that could prevent some epiphyte species from colonizing them (Fig. 1). In this second scenario, host plant evolutionary distinctiveness would be positively associated with the PD of its epiphyte community. Finally, our third hypothesis, the determinants of host suitability for colonization by epiphytes might be unrelated to its phylogenetic history, such that no significant relationship would be found between host tree evolutionary distinctiveness and the PD of its epiphyte community. It might not be evolutionary history that is important, and there is very little evidence for host specificity in epiphytes, suggesting that host characteristics are affecting epiphyte communities more. Studies found rather little host specificity for epiphytes (Wagner, Mendieta-Leiva & Zotz, 2015); thus it is also predicted that phylogenetic history might also not be of importance. To the best of our knowledge, none of these scenarios have explicitly been tested before.

## Materials & Methods

A database of epiphytes (‘true’ epiphytes, hemiepiphytes and nomadic vines sensu (Zotz, 2013a; Zotz et al., 2021a) and their host plants (*e.g.*, trees, palms, shrubs, tree ferns) was obtained in October 2021 from the Epiphyte Inventory Database (EpIG-DB), a database of vascular epiphyte assemblages in the Neotropics (Mendieta-Leiva et al., 2020). EpIG-DB contains epiphyte assemblages from 18,148 relevés sampled within 687 forest plots across the Neotropics. We extracted all epiphyte (2,890 species) and host (1,551 species) related information for 4,441 species from the EpIG-DB database. The information in the extracted database included: sample location (incl. latitude and longitude), host species, diameter at breast height (DBH) and total height, and epiphyte species. We corrected manually any taxonomic issues using Zotz et al. (2021a); Zotz et al. (2021b), the Plant List (http://www.theplantlist.org/), and Tropicos (http://www.tropicos.org/). We omitted records only identified to the family level, but kept the species identified to the genus level. This compilation generated an incidence matrix, reporting which epiphytes are present on each host plant. Given that some host and epiphyte names appeared more than once in the original dataset, we combined the rows/columns in the incidence matrix that included the same species. For the other characters used here, we averaged them across multiple records of the same species.

Although considerable advances have been made in recent decades, we are still far from a complete species-level phylogeny of all plants, especially with vascular epiphytes. Consequently, plant studies often used family-level phylogenetic information, particularly through the use of the ‘phylomatic’ tool (Webb & Donoghue, 2004). In this study, we employed an alternative approach that used phylogenetic imputation to generate alternative, species-level phylogenies of the species in our dataset known as the Taxonomic Addition for Complete Trees (TACT; Chang, Rabosky & Alfaro (2020)). We began by establishing a backbone tree using the phylogenies of Qian & Zhang (2014) for seed plants and Testo & Sundue (2016) for ferns. In both phylogenies, we pruned all families that were not present in our dataset. We had to exclude 63 species (Table S1) of plants from the incidence matrix described above, given that their families were not present in any of the backbones. We joined the two trees by placing the phylogeny of the seed plants at the tips of the ferns tree corresponding to the seed plants, such that the fern tree includes some seed plants as the outgroup. We then used taxonomic information to place species within genera and genera within families based on a model of lineage diversification (see Chang, Rabosky & Alfaro (2020) for more details). We generated 100 alternative phylogenetic trees that included all 4,441 species in our dataset. All analyses were repeated with this set of phylogenetic trees to account for phylogenetic uncertainty. All analysis were performed using R 4.1.1 (R Core Team, 2021).

There are many alternative metrics to describe phylogenetic distinctiveness and diversity (Vellend et al., 2011). For host plants, we chose the ’species evolutionary history’ (SEH) metric of Redding & Mooers (2006), instead of using its modification known as evolutionary distinctiveness (ED) of Redding et al. (2008). Both seek to assess the portion of a phylogenetic diversity in a tree attributable to any particular species, but they differ in their calculations: in SEH, shared branches are apportioned equally among daughter clades (‘equal splits’), whereas in ED, shared branches are apportioned equally among descendant species (‘fair proportions’). However, simulations have shown that SEH and ED are highly correlated (Vellend et al., 2011), therefore we only used SEH here. For the associated epiphytes, we used the mean pairwise distance (MPD), as it can be more sensitive under some conditions, to detect non-random community assembly than the alternative, distance-based metrics (Vellend et al., 2011). We calculated SEH for the host plants and MPD for the epiphytes using the evol.distinct and mpd functions, respectively, in the R package ‘picante’ 1.8.2 (Kembel et al., 2010).

We used phylogenetic generalized least squares regression (PGLS) to test the relationships between the host and epiphyte metrics of the available data for all species, namely: (i) SEH of the host plants as the predictor variable and MPD of the epiphytes present in each host plant as the response variable; (ii) SEH of the host plants as the predictor variable and the number of epiphyte species in the host plant as the response variable; (iii) mean latitudinal position of host plants as the predictor variable and MPD of the epiphytes as the response variable; and (iv) mean DBH of the host plants as the predictor variable and MPD of the epiphytes as the response variable. PGLS analyses were performed using the pgls function in the R package ’caper’ 1.0.1 (Orme et al., 2018), with the phylogenetic signal of the residuals being estimated during the regression. We chose SEH and MPD metrics because simulations demonstrated that they were robust to incomplete taxonomic sampling (data not shown). In addition, we also evaluated the phylogenetic signal of MPD of the epiphytes in each host, mean latitudinal position and mean DBH of the host plants, both using Pagel’s *λ* (Pagel, 1999) and Blomberg, Garland Jr & Ives (2003) K with phylosig function in the R package ’phytools’ 0.7–90 (Revell, 2012). All variables with a skewed distribution were log-transformed prior to the analyses. All R scripts and the database have been provided within the supplementary material.

The R script for our analysis, all relevant datafiles and a supporting analysis metadata file can be found here: https://github.com/fernandacaron/epi_evol (https://doi.org/10.5281/zenodo.7714935).

## Results

**Figure 2:**
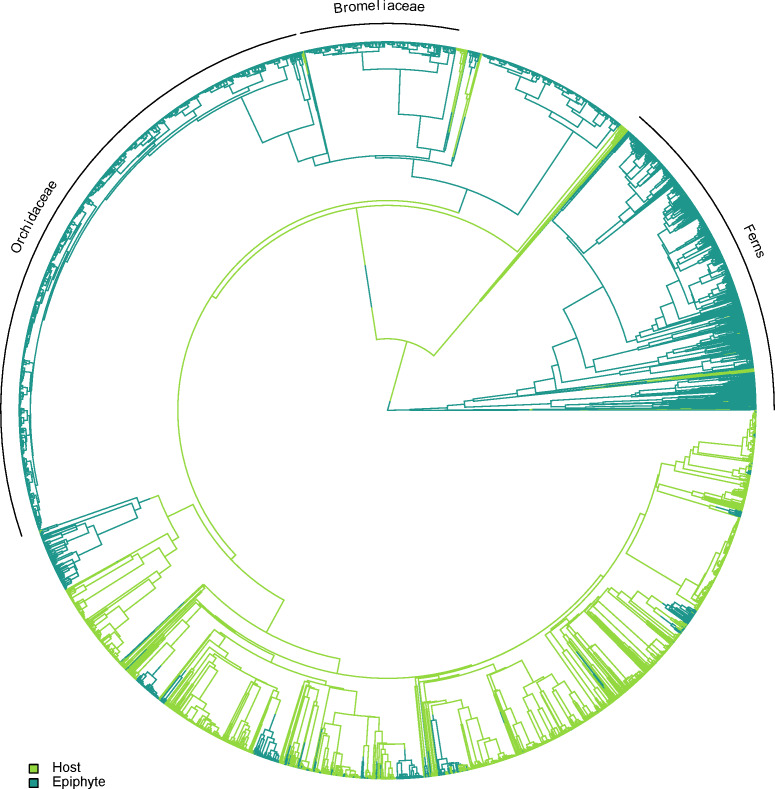
One of the alternative topologies representing the phylogenetic relationships of vascular epiphytes and their hosts in our dataset. Three major epiphytes groups are highlighted, namely ferns, bromeliads and orchids.

Epiphytes are found in several independent lineages across the plant tree of life, with only a small number of potential reversals back to the terrestrial habit. A visual example of one topology generated using TACT is shown in Fig. 2.

**Figure 3:**
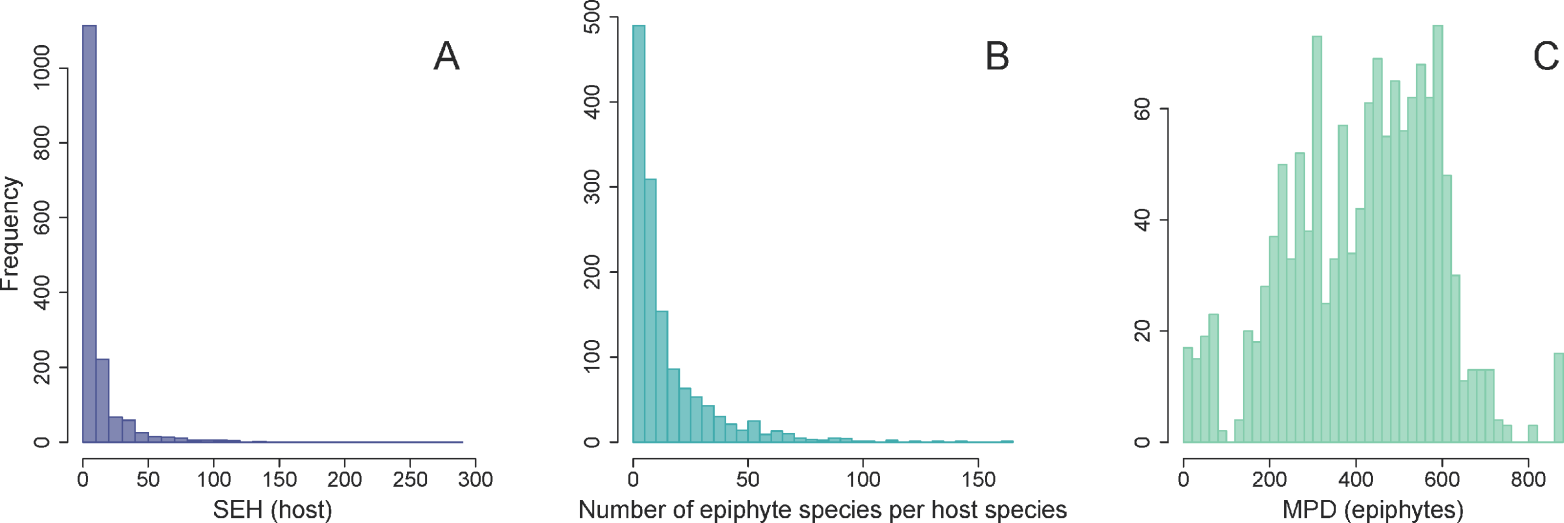
Frequency distributions of host and epiphytes metrics. Frequency distribution of host SEH (species evolutionary history) (A), the number of vascular epiphyte species associated with each host (B), and their associated MPD (mean phylogenetic distance) (C).

The distribution of SEH was highly skewed across host plants (Fig. 3A). Most species had low to intermediate levels of SEH, with the exception of a few lineages such as *Cupressus lusitanica* Mill. and the tree fern *Dicksonia sellowiana* Hook. that showed considerably higher SEH. We found that the number of epiphyte species per host species was highly skewed (Fig. 3B), varying from a minimum of one species per host species, to a maximum of 163 in the case of *Alchornea triplinervia* (Spreng.) Müll. Arg.. On the other hand, the distribution of epiphyte MPD was largely unimodal (Fig. 3C).

**Figure 4:**
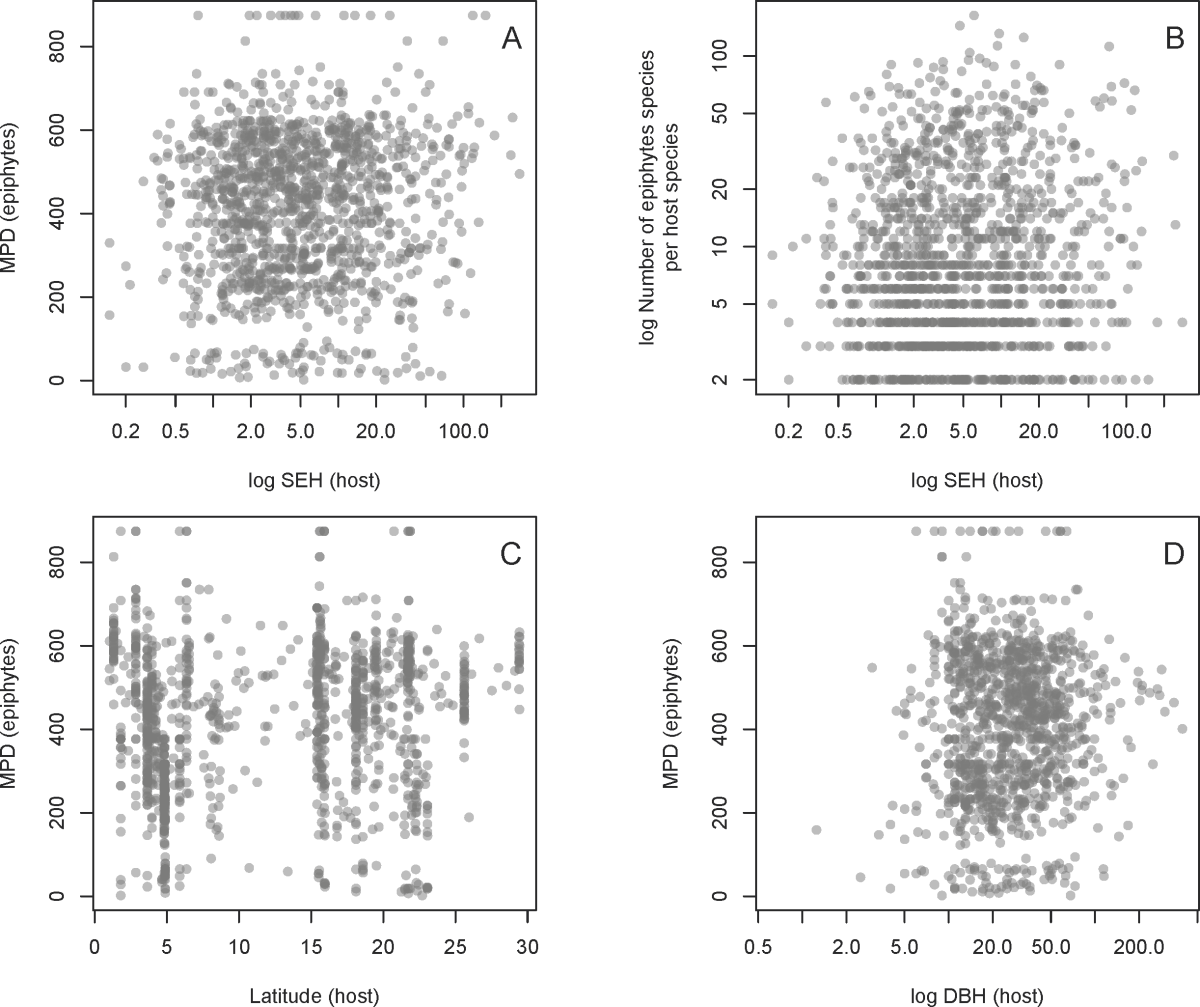
Relationships between the host and epiphyte metrics. Relationships of host SEH (species evolutionary history) against MPD (mean phylogenetic distance) (A) and the number of vascular epiphyte species per host (B), as well as two potential confounding effects on MPD, namely absolute latitude (C) and the logarithm of host diameter at breast height (DBH) (D). See text for details.

We found no evidence for a relationship between host SEH and the phylogenetic diversity (as measured by MPD) of the host’s associated epiphyte species (Fig. 4, Table 1). This result is unlikely to be due to the confounding effects of latitude, host DBH, or the total number of epiphyte species associated with each host species (Fig. 4, Table 1). One might suppose that the absence of a relationship between host SEH and the epiphyte MPD could be because the latter might be evolutionarily labile. However, there is significant phylogenetic signal in epiphyte MPD, as well as in latitude and host DBH (Table 2).

**Table 1:** Phylogenetic generalized least squares (PGLS) analyses of the proposed models. Response variables correspond to the number and the mean pairwise distance (MPD) of epiphytes in each host, and predictor variables correspond to species evolutionary history (SEH), absolute latitude, and diameter at breast height (DBH) of host plants.

**Response**	**Predictor**	**N**	**Slope**	**SE**	**t**	**P**	**R** ^**2**^
log(number of epiphyte species)	log(SEH)	1,347	−0.01 (−0.4–0.6)	0.1 (0.1–0.8)	−0.06 (−2.9–5.6)	0.6 (0–0.9)	0 (0-0.02)
MPD	log(SEH)	1,347	3.8 (−65.9–99.2)	22.5 (14.1–135.1)	0.1 (−3.8–7.1)	0.5 (0–0.9)	0 (0–0.07)
	Latitude	1,347	1.9 (−17.3–27.2)	0.7 (0.1–3.6)	2.8 (−41.9–133.2)	0 (0–0.9)	0.02 (0–0.9)
	DBH	1,160	26.8 (−330.5–833.5)	6.9 (2.1–27.6)	3.8 (−41.02–81.9)	0 (0–0.9)	0.03 (0–0.9)

**Notes.**

Estimates are provided as medians and ranges across 100 alternative topologies.

**Table 2:** Phylogenetic signal of mean pairwise distance (MPD) of the epiphytes occurring in each host, and latitude and diameter at breast height (DBH) of host plants.

**Trait**	**N**	*λ*	**logL**	**logL0**	**P**	**K**	**P**
MPD	1,347	0.2 (0.2–0.3)	−8,803.3 (−8,851.8–8,698.4)	−8,817.5 (−8,867.5–8,714.1)	0 (0–0)	0.002 (0–0.004)	0.02 (0.001–0.9)
Latitude	1,553	0.6 (0.6–0.7)	−5,296.8 (−5,305.7–5,286.7)	−5,430.2 (−5,430.2–5,430.2)	0 (0–0)	0.002 (0–0.004)	0.001 (0.001–0.8)
DBH	1,339	0.4 (0.3–0.5)	−6,577.5 (−6,583.1–6,571.2)	−6,616.2 (−6,616.2–6,616.2)	0 (0–0)	0.001 (0–0.003)	0.3 (0.009–0.9)

**Notes.**

Estimates are provided as medians and ranges across 100 alternative topologies.

## Discussion

Our results demonstrate that the MPD of the vascular epiphyte species associated with a given host plant species is largely independent of its evolutionary distinctiveness. Before exploring the implications of these results, it is important to revisit the rationale of the tested hypotheses to ensure that they are reasonable. First, we hypothesized a negative relationship between host ED and epiphyte PD, given that a host tree that is evolutionarily unique (*i.e.,* on a long phylogenetic branch) may have uncommon characteristics that could make it less likely to be colonized by epiphytes. This rationale is based on the widespread observation that functional traits are often well approximated by a Brownian-like process, with trait variance evolving at a relatively constant rate over time. One can envision a geographical region in which two sister groups of host plants are found—one with a single species and the other that is species-rich. Under a scenario of relatively constant rate of host plant trait evolution, the members of the second group would tend to resemble each other, causing their functional traits to be more prevalent in that region. As epiphytes become adapted to their local environments, they would tend to be exposed most often to the traits found in the most species-rich host group. Alternatively, the sister group with only one species could display relatively uncommon traits, leading to some level of filtering of potential epiphytes, leading to low PD. Second, we might envision a scenario in which an evolutionarily unique host plant would have been available for colonization for a long time, allowing ample time to be colonized by epiphytes, whereas relatively young lineages could show novel trait combinations that could hamper epiphyte colonization. If true, one could predict a positive association between host plant evolutionary distinctiveness and the PD of its epiphyte community Although this scenario would be more easily understood if evolutionarily distinct lineages show relatively low rates of evolution (as in the case of many relictual lineages), that is not necessarily a requirement. For instance, epiphytes could more easily track the evolution of a single evolutionary lineage than a set of closely related lineages that differ to varying degrees, due to potential trade-offs when becoming adapted to phenotypically variable host plants. Finally, our third hypothesis—the determinants of host suitability for colonization by epiphytes might be unrelated to its phylogenetic history, such that no significant relationship would be found between host tree evolutionary distinctiveness and the PD of its epiphyte community—serves as a null model in relation to the first two hypotheses. Although in hindsight this reasoning might sound simplistic, it is usually worth starting simple and adding complexity to hypotheses as the evidence requires it.

Given the opportunistic nature of commensal communities, one might interpret our results as an indication that interspecific host variation in their epiphyte PD is largely due to neutral/stochastic mechanisms unrelated to processes at large evolutionary timescales as predicted by our third hypothesis. Although we do not reject the notion of the important role of neutral mechanisms, it is important to keep in mind that there was substantial evidence for phylogenetic signals in epiphyte PD across different host species (Table 2). In other words, if a host plant has a phylogenetically diverse set of associated epiphytes, closely related host species are also likely to show high epiphyte PD. Some studies have uncovered proximate mechanisms that might influence the presence of different epiphyte species, such as habitat conditions, availability of propagules, their dispersal characteristics and requirements for seedling establishment (Cascante-Marín et al., 2006; Burns, 2007; Ceballos, Chacoff & Malizia, 2016; Francisco et al., 2018; Francisco et al., 2019; Zotarelli et al., 2019), as well as the physical characteristics of the host plants (Sanger & Kirkpatrick, 2014). One might therefore hypothesize that these properties of host plants might predict epiphyte PD in a way that is independent of host evolutionary distinctiveness. Although we concede that these arguments are still highly speculative, the scarcity of other empirical studies on drivers of epiphyte PD still precludes more precise predictions. However, we hope that our results will stimulate more research on commensal PD and its determinants.

Although we present the most comprehensive test of a determinant of epiphyte PD to date, there are a few important caveats. First, although the plant phylogenies we used included many species, they were far from complete. However, although incomplete taxonomic sampling might indeed affect estimates of SEH, the missing species are likely to occur in specific geographical regions that are far from the occurrence records included in our dataset, and therefore would not be particularly relevant, given the rationale behind the hypotheses tested in our study (Fig. S1). Another consequence of incomplete taxon sampling for estimating SEH is that estimates from incomplete trees are necessarily lower than those of complete trees because of the way this statistic is computed. However, estimates from pruned trees are still significantly correlated to those of complete trees (rho = 0.46, *p* < 2.2e−16), although imperfectly, so that our conclusions should be revisited once improved phylogenetic knowledge is improved over time. Finally, although our broad-scale analysis did not find a relationship between the MPD of the vascular epiphyte species and the evolutionary distinctness of host plant species, it might still take place at smaller geographical scales.

Since Burns (2007), many studies investigate the commensal relationship between epiphytes and its host species from tropical to temperate forests. Differently from parasitic relationship, such as those from mistletoes and lianas (Blick & Burns, 2009), the epiphytic commensal ones showed nested and low specialization, based only on the trees abundance (or density), size (height and diameter) and/or bark traits (wood density and bark texture) (Burns, 2007; Silva et al., 2010; Sáyago et al., 2013; Ceballos, Chacoff & Malizia, 2016; Francisco et al., 2018; Francisco et al., 2019; Zotarelli et al., 2019; Francisco et al., 2021). These studies suggest that the epiphytes are selecting its host trees for colonization more based on specific host traits, such as area/habitat opportunity, a greater number of microenvironment and the time available for colonization, rather than specific host species. Although Callaway et al. (2002) and Wagner, Mendieta-Leiva & Zotz (2015) suggested that phorophyte specificity could be more evident in suboptimal habitats, some studies suggested that even in these habitats this relationship showed low specificity. In Brazilian Inselbergs, despite the low diversity of potential phorophytes, instead of species preferences, Francisco et al. (2018) found that epiphyte richness was influenced by the phorophyte diameter.

As mentioned above, the overall influence of the tree host traits on epiphyte assemblage diversity is complex and specific. Tree size (*e.g.*, DBH (Zotz & Schultz, 2008); height (Flores-Palacios & García-Franco, 2006; Fayle et al., 2009)) for example, showed a positive influence on epiphyte species richness and abundance in biomes with higher epiphyte diversity. Larger host trees suggest more area available to epiphyte establishment (Gradstein et al., 2003; Werner et al., 2012), reducing the epiphyte competition, and consequently harboring more epiphyte species. Also, tallest trees could have a vertical stratification of environmental conditions on the tree canopy (*e.g.*, light, moisture) (Murakami et al., 2022), providing a highest diversity of microhabitats for a wider range of epiphytes species (Krömer, Kessler & Gradstein, 2007; Fayle et al., 2009; Dislich & Mantovani, 2016). However, in disturbed habitats (*e.g.*, pasture, secondary forest), showing lower epiphyte diversity, few dominant drought-tolerant species, such as atmospheric *Tillandsia* or poikilohydric polypodioid ferns, are able to occur on host trees (Hietz, Buchberger & Winkler, 2006; Larrea & Werner, 2010; Poltz & Zotz, 2011; Krömer, García-Franco & Toledo-Aceves, 2014; Einzmann & Zotz, 2017; Elias et al., 2021; Trejo-Cruz et al., 2021). Even tree size showing a positive influence on epiphyte assemblage species richness as in the forest, other traits (*e.g.*, bark rugosity) are equally, or even more important, to the host harbor higher epiphyte diversity (Poltz & Zotz, 2011). Elias et al. (2021) showed that larger pasture trees tend to harbor more epiphyte species and individuals. However, only large hosts with rugose bark, harbored drought-tolerant epiphyte species and more forest specialist epiphytes compared to smaller trees.

Our study suggests that neutral processes might dominate phylogenetic community assemblages of epiphytes, and is consistent with previous studies (Ceballos, Chacoff & Malizia, 2016; Zotarelli et al., 2019). Dispersal limitation, among neutral processes (random extinction and speciation, and migration), is an important factor in community assembly, despite variation in environmental conditions (Hubbell, 2006). The commensalistic relationship involving abundance and phorophyte traits (*e.g.*, height, diameter and bark types) suggest that the random encounters of individuals (regardless of the species to which they belong) concurring in space and time, gradually built up these communities (Sáyago et al., 2013).

Although significant phylogenetic signal is not uncommon on mutualistic and antagonistic interactions (Rezende, Jordano & Bascompte, 2007a; Rezende et al., 2007b; Gómez, Verdú & Perfectti, 2010; Eklöf et al., 2012), we did not find evidence for it in our analysis of epiphyte-host interactions. Two other studies on commensal interactions, but within specific families, such as epiphytic orchid- (Brazil; Silva et al. (2010)) and bromeliad- (Mexico; Sáyago et al. (2013)) host tree networks, also did not find evidence for phylogenetic signal. According to Thompson (2005), natural selection acting on epiphyte-host tree networks may not tend to favor convergence of traits among the interacting species, only among epiphytes. Although Sáyago et al. (2013) suggested that the lack of support for phylogenetic signal could be due to the use of small phylogenies, that is unlikely to be the case in our dataset. Nevertheless, we found evidence for phylogenetic signal in epiphyte PD across different host species. It is important to note that the phylogenetic signal in epiphyte PD might be due to phylogenetic autocorrelation at parts of the tree, such as (i) within or among food web compartments (Rezende et al., 2009), (ii) according to different species roles in food webs (Stouffer et al., 2012) and/or (iii) within particular clades (Gómez, Verdú & Perfectti, 2010).

## Conclusions

In conclusion our study demonstrated that the PD of an epiphyte community is unrelated to the host evolutionary distinctiveness. This could imply that mechanisms operating at ecological timescales might be more important in epiphyte community assembly and exploring those mechanisms might shed more light of the local, regional, global, and commensal biodiversity patterns. To the best of our knowledge, this is the first study that underscores the utility of using community phylogenetics and phylogenetic diversity to try and understand community assembly mechanisms.

##  Supplemental Information

10.7717/peerj.15500/supp-1 Supplemental Information 1 Map of the location of samples that were included in the analysis

10.7717/peerj.15500/supp-2 Supplemental Information 2 Species that were excluded from the incidence matrix

## Additional Information and Declarations

### Data Availability

The following information was supplied regarding data availability: The R script for our analysis, all relevant datafiles and a supporting analysis metadata file are available at GitHub and Zenodo:
https://github.com/fernandacaron/epi_evol. Sven Batke (corresponding author). (2023). Phylogenetic diversity and the structure of host-epiphyte interactions. Zenodo. https://doi.org/10.5281/zenodo.7714935.
