# Supplementary figures and images for: Phylogenetic diversity and the structure of host-epiphyte interactions across the Neotropics
Source: PeerJ. 2023 Jun 19;11:e15500. doi: 10.7717/peerj.15500 (PMC10286801; doi:10.7717/peerj.15500)

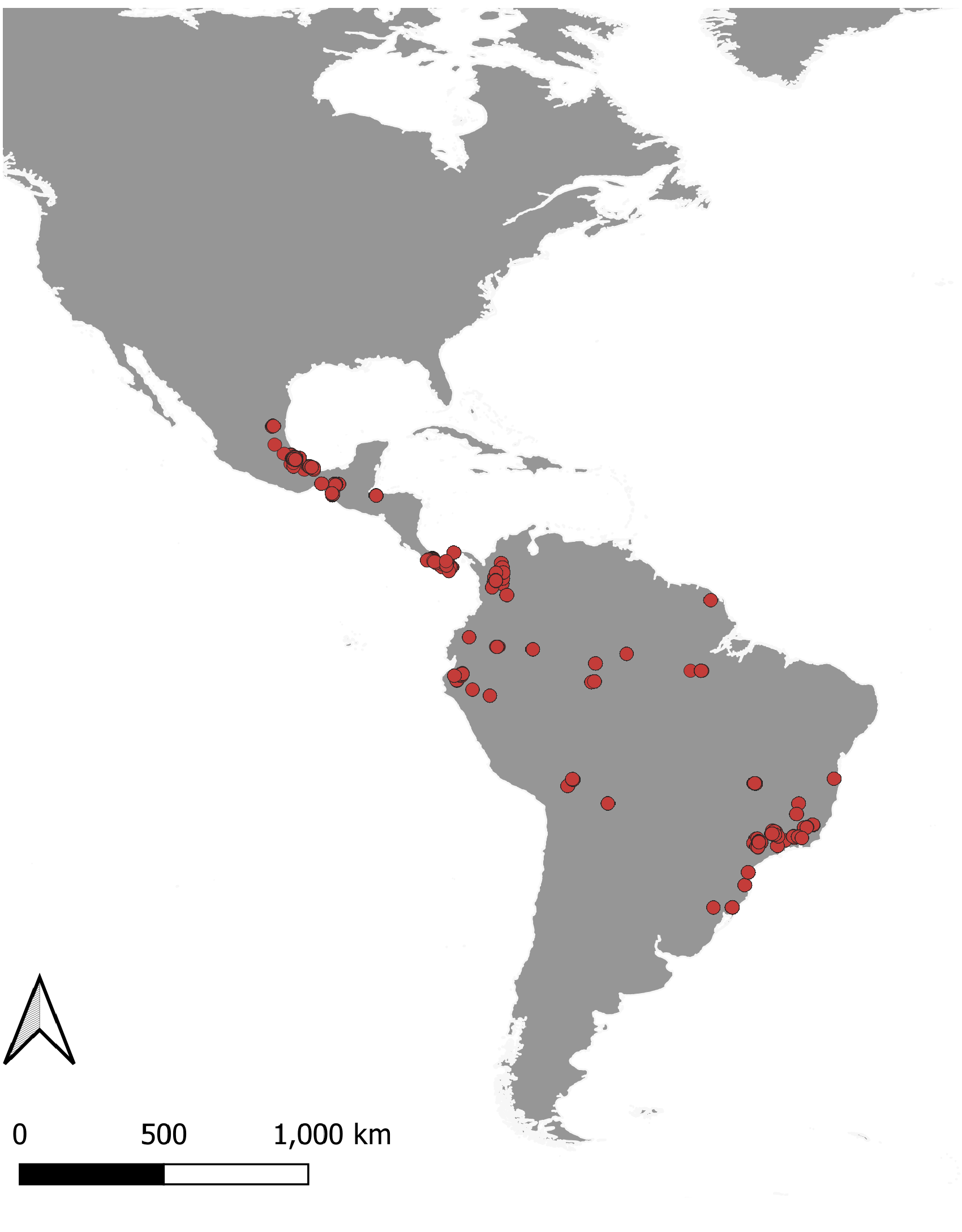

Supplement: Supplemental Information 1 [file peerj-11-15500-s001.png]
