# Supplementary material for: Phylogenetic diversity and the structure of host-epiphyte interactions across the Neotropics
Source: PeerJ. 2023 Jun 19;11:e15500. doi: 10.7717/peerj.15500 (PMC10286801; doi:10.7717/peerj.15500)
Supplement: Supplemental Information 2 [file peerj-11-15500-s002.docx]

**Supporting Information**

**Table S1.** Species that were excluded from the the incidence matrix. See text for further detail.

| **Family** | **Species** | **Type** |
| --- | --- | --- |
| Cordiaceae | *Cordia* ESsp.1 | Host |
| Cordiaceae | *Cordia americana* | Host |
| Cordiaceae | *Cordia ecalyculata* | Host |
| Cordiaceae | *Cordia alliodora* | Host |
| Cordiaceae | *Cordia* HEsp1 | Host |
| Cordiaceae | *Cordia* HEsp3 | Host |
| Cordiaceae | *Cordia megalantha* | Host |
| Cordiaceae | *Cordia exaltata* | Host |
| Cordiaceae | *Cordia fallax* | Host |
| Cordiaceae | *Cordia* MA1009 | Host |
| Cordiaceae | *Cordia* sp02 | Host |
| Cordiaceae | *Cordia sellowiana* | Host |
| Holocalyx | *Holocalyx balansae* | Host |
| Homalopetalum | *Homalopetalum pumilio* | Epiphyte |
| Leguminosae | *Cynometra bauhinifolia* | Host |
| Leguminosae | *Erythrina berteroana* | Host |
| Leguminosae | *Erythrina falcata* | Host |
| Leguminosae | *Erythrina fusca* | Host |
| Lycopodiaceae | *Huperzia reflexa* | Epiphyte |
| Lycopodiaceae | *Huperzia dichaeoides* | Epiphyte |
| Lycopodiaceae | *Huperzia fontinaloides* | Epiphyte |
| Lycopodiaceae | *Huperzia biformis* | Epiphyte |
| Lycopodiaceae | *Huperzia heterocarpon* | Epiphyte |
| Lycopodiaceae | *Huperzia taxifolia* | Epiphyte |
| Lycopodiaceae | *Huperzia lindenii* | Epiphyte |
| Lycopodiaceae | *Huperzia aqualupiana* | Epiphyte |
| Lycopodiaceae | *Huperzia* spPasP1 | Epiphyte |
| Lycopodiaceae | *Huperzia pringlei* | Epiphyte |
| Lycopodiaceae | *Huperzia pithyoides* | Epiphyte |
| Lycopodiaceae | *Huperzia cuernavacensis* | Epiphyte |
| Lycopodiaceae | *Huperzia orizabae* | Epiphyte |
| Lycopodiaceae | *Huperzia myrsinites* | Epiphyte |
| Lycopodiaceae | *Huperzia capillaris* | Epiphyte |
| Lycopodiaceae | *Huperzia* sp 1WerTr | Epiphyte |
| Lycopodiaceae | *Huperzia erectifolia* | Epiphyte |
| Lycopodiaceae | *Huperzia wilsonii* | Epiphyte |
| Lycopodiaceae | *Lycopodium* JWsp1 | Epiphyte |
| Lycopodiaceae | *Phlegmariurus ericifolius* | Epiphyte |
| Lycopodiaceae | *Phlegmariurus pithyoides* | Epiphyte |
| Lycopodiaceae | *Phlegmariurus phylicifolius* | Epiphyte |
| Lycopodiaceae | *Phlegmariurus pringlei* | Epiphyte |
| Lycopodiaceae | *Phlegmariurus cuernavacensis* | Epiphyte |
| Lycopodiaceae | *Phlegmariurus quadrifariatus* | Epiphyte |
| Lycopodiaceae | *Phlegmariurus heterocarpon* | Epiphyte |
| Lycopodiaceae | *Phlegmariurus fontinaloides* | Epiphyte |
| Lycopodiaceae | *Phlegmariurus filiformis* | Epiphyte |
| Lycopodiaceae | *Phlegmariurus flexibilis* | Epiphyte |
| Lycopodiaceae | *Phlegmariurus acerosus* | Epiphyte |
| Lycopodiaceae | *Phlegmariurus* MA1018 | Epiphyte |
| Lycopodiaceae | *Phlegmariurus linifolius* | Epiphyte |
| Meliosma | *Meliosma corymbosa* | Host |
| Meliosma | *Meliosma herbertii* | Host |
| Meliosma | *Meliosma sellowii* | Host |
| Meliosma | *Meliosma alba* | Host |
| Meliosma | *Meliosma* sp1 | Host |
| Meliosma | *Meliosma* sp2 | Host |
| Peraceae | *Pera glabrata* | Host |
| Petiveriaceae | *Gallesia integrifolia* | Host |
| Selaginellaceae | *Selaginella schizobasi* | Epiphyte |
| Selaginellaceae | *Selaginella microstachya* | Epiphyte |
| Selaginellaceae | *Selaginella extensa* | Epiphyte |
| Viscoideae | Viscoideae sp 4WerTr | Epiphyte |
| Viscoideae | Viscoideae sp 2WerTr | Epiphyte |
